# Socioeconomic Deprivation and the Incidence of 12 Cardiovascular Diseases in 1.9 Million Women and Men: Implications for Risk Prediction and Prevention

**DOI:** 10.1371/journal.pone.0104671

**Published:** 2014-08-21

**Authors:** Mar Pujades-Rodriguez, Adam Timmis, Dimitris Stogiannis, Eleni Rapsomaniki, Spiros Denaxas, Anoop Shah, Gene Feder, Mika Kivimaki, Harry Hemingway

**Affiliations:** 1 Department of Epidemiology and Public Health and Farr Institute of Health Informatics Research, University College London, London, United Kingdom; 2 National Institute for Health Research Biomedical Research Unit, Barts and the London School of Medicine and Dentistry, London, United Kingdom; 3 Centre for Academic Primary Care, School of Social and Community Medicine, Bristol, United Kingdom; 4 Department of Epidemiology and Public Health, University College London, London, United Kingdom; The George Institute for Global Health, Australia

## Abstract

**Background:**

Recent experimental evidence suggests that socioeconomic characteristics of neighbourhoods influence cardiovascular health, but observational studies which examine deprivation across a wide range of cardiovascular diseases (CVDs) are lacking.

**Methods:**

Record-linkage cohort study of 1.93 million people to examine the association between small-area socioeconomic deprivation and 12 CVDs. Health records covered primary care, hospital admissions, a myocardial infarction registry and cause-specific mortality in England (CALIBER). Patients were aged ≥30 years and were initially free of CVD. Cox proportional hazard models stratified by general practice were used.

**Findings:**

During a median follow-up of 5.5 years 114,859 people had one of 12 initial CVD presentations. In women the hazards of all CVDs except abdominal aortic aneurysm increased linearly with higher small-area socioeconomic deprivation (adjusted HR for most vs. least deprived ranged from 1.05, 95%CI 0.83–1.32 for abdominal aortic aneurysm to 1.55, 95%CI 1.42–1.70 for heart failure; I^2^ = 81.9%, τ^2^ = 0.01). In men heterogeneity was higher (HR ranged from 0.89, 95%CI 0.75–1.06 for cardiac arrest to 1.85, 95%CI 1.67–2.04 for peripheral arterial disease; I^2^ = 96.0%, τ^2^ = 0.06) and no association was observed with stable angina, sudden cardiac death, subarachnoid haemorrhage, transient ischaemic attack and abdominal aortic aneurysm. Lifetime risk difference between least and most deprived quintiles was most marked for peripheral arterial disease in women (4.3% least deprived, 5.8% most deprived) and men (4.6% least deprived, 7.8% in most deprived); but it was small or negligible for sudden cardiac death, transient ischaemic attack, abdominal aortic aneurysm and ischaemic and intracerebral haemorrhage, in both women and men.

**Conclusions:**

Associations of small-area socioeconomic deprivation with 12 types of CVDs were heterogeneous, and in men absent for several diseases. Findings suggest that policies to reduce deprivation may impact more strongly on heart failure and peripheral arterial disease, and might be more effective in women.

## Introduction

A recent randomized social experiment has provided evidence of the association between neighbourhood poverty and long-term poor physical and mental health [1], as well as increased prevalence of cardiovascular risk factors such as obesity and diabetes [2]. This observation suggests that socioeconomic deprivation can be considered as a modifiable risk factor for cardiovascular disease (CVD) and that its ill effects might be reversible. Area deprivation provides information on living circumstances, which are not captured by individual-level information. For instance neighbourhoods may influence the life chances of individuals through their effects on achieved education, occupation, income, services and resources availability. And living in disadvantaged neighbourhoods may be one of the mechanisms leading to adverse health outcomes in persons with low socioeconomic status. This might have important implications for reduction in health inequalities. Despite improvements in population risk factors in recent years and reductions in mortality from coronary heart disease, socioeconomic gradients in health status have persisted or worsened [3]–[5]. In the context of the current financial crisis, health inequalities are expected to increase further. Evidence to guide policy, for instance to identify patient groups likely to benefit most from prevention strategies, or to inform how aggressively cardiovascular risk factors should be managed in different groups, is therefore important to ensure adequate management of patients and resource allocation.

The risk of acute myocardial infarction, stroke and coronary death has been shown to increase with higher levels of individual and community deprivation [6]–[14]. However, because the incidence of these diseases is rapidly declining [15], [16] these conditions now account for less than a third of cardiovascular presentations. In focusing on these outcomes, researchers have left unanswered questions about other types of common CVDs and have provided an incomplete picture of how socioeconomic deprivation is associated with disease development. For example, estimates of associations with angina, sudden cardiac death [17], [18], peripheral vascular disease [19]–[22] or specific types of stroke [15] are uncommon. Information is also scarce about the potential for gender, age or comorbidities to modify these associations. The incidence of myocardial infarction and coronary death increases with greater deprivation in women and men [6], and is lower in older age groups [12]. However, sex-specific effect sizes may differ and the extent to which incidence decreases with age for different CVDs is unknown.

In the United Kingdom availability of linked patient electronic health records covering primary, secondary and specialist care and integration of postcode based indicators of socioeconomic deprivation into electronic health records offer a unique opportunity to examine heterogeneity in small-area socioeconomic inequalities across different CVDs, by sex and age. These deprivation indicators calculated at small-area level are used as a proxy for material deprivation and their association with coronary heart disease has been shown to be independent from measures of socioeconomic position such as income, education or occupation [23]. They are also included in existing cardiovascular risk prediction scores used in clinical practice to aid preventive treatment decision [24]–[27].

In the present study we have used a large scale, contemporary cohort based on linked electronic health records to investigate social inequalities in cardiovascular health. Specifically, we have: (i) estimated lifetime risks of the 12 most common initial and first event presentations of CVDs in women and men in relation to level of small-area socioeconomic deprivation; (ii) assessed and compared associations (hazard ratios) of small-area socioeconomic deprivation across different CVDs, and (iii) examined whether these associations are modified by age, current smoking or co-morbidities.

## Methods

### Study Population

A cohort of 1,937,360 patients identified amongst individuals who were registered in the general practices contributing with data to the CALIBER programme (Cardiovascular disease research using LInked Bespoke studies and Electronic health Records) [28], between January 1997 and March 2010 was studied. Patients were included in the analysis regardless of whether or not they had a recorded consultation during this time period. The CALIBER programme was established to provide access to longitudinal data of multiple linked electronic health records (EHR) sources by establishing a common data model with reproducible EHR phenotypes and meta-data. Diagnosis codes and endpoints in CALIBER have been validated by independent groups [29]. Patient electronic medical records were linked across four data sources: the Clinical Practice Research Datalink (CPRD), formerly known as the General Practice Research Database [30]; the Myocardial Ischaemia National Audit Project disease registry (MINAP) [31]; Hospital Episodes Statistics (HES); and the national death registry (Text S1). In the United Kingdom nearly all citizens are registered with a general (primary care) practitioner [32]. CPRD provides primary care data on medical history, clinical diagnoses, anthropometric measures, health behaviors, laboratory tests, medical procedures and prescriptions, coded using the Read clinical coding system. Patients in the subset of linkable practices were representative of the whole CPRD as evidenced by a number of measures including demographics (e.g. age, socioeconomic deprivation), prescribing and comorbidity [33]. CPRD provides a representative dataset of the UK primary care setting [30], and has been extensively used and validated for epidemiological research [29]. MINAP is a national registry of patients admitted to hospital with acute coronary syndromes. HES provides information on diagnosis (coded with the ICD-10) and medical procedures related to all elective and emergency hospital admissions across all National Health Service hospitals in England. The validity of the CALIBER cohort for research into CVDs is supported by our recent demonstration of associations between blood pressure and the same twelve diseases reported here; replicating known associations (heart attack and stroke) and extending knowledge where cohort literature has been sparse (e.g. abdominal aortic aneurysm, peripheral arterial disease) [34]. Inclusion criteria for the analysis were age ≥30 years, one year or more of follow-up, and free of CVD at baseline (Figure S1).

### Socioeconomic deprivation

The level of socioeconomic deprivation was measured with the index of multiple deprivation (IMD) 2007 calculated at lower layer super output area level (small geographical areas, 23 482 defined in England with an average population of 1500 people), that had been linked by a third trust party using the patient postcode of residence recorded in CPRD [35]. This measure of community socioeconomic status is a composite indicator commonly used in the United Kingdom that has been created by the Office of National Statistics using census postcode data. It is calculated combining 38 indicators of seven domains of deprivation: income, employment, health and disability; education, skills and training; barriers to housing and services, crime, and living environment [36], [37]. The IMD is the combined sum of the weighted, exponentially transformed domain ranks of the domain score. Explicit pre-defined weights are used (22.5% income, 22.5% employment, 13.5% health and disability, 13.5% education, skills and training, 9.3% barriers to housing and services, 9.3% crime, and 9.3% living environment). Because IMD is a non-linear measure of community socioeconomic status, for the analysis patients were categorised into quintiles of small-area deprivation, where the first quintile indicated the least deprived and the fifth quintile the most deprived group [36], [37].

### Covariates

Covariates considered in the analysis were: sex, age, ethnicity, diabetes mellitus, smoking status, body mass index, systolic blood pressure, total and high-density lipoprotein cholesterol, and medication use (blood pressure lowering drugs, statins, oestrogen oral contraceptives and hormone replacement therapy). The most recent measurement (or prescription) recorded in CPRD up to one year before study entry was used to define baseline covariates. Patients were defined as diabetic if they had a diagnosis or a prescription of hypoglycemic drugs prior to baseline. Definitions of covariates can be found at https://www.caliberresearch.org/portal/.

### Endpoints

The primary endpoints for the analysis were the initial presentation of fatal and non-fatal CVD identified across the aforementioned data sources. CVD presentations studied were: stable angina, unstable angina, myocardial infarction, heart failure, a composite of ventricular arrhythmia, cardioversion, cardiac arrest or sudden cardiac death (CA-SCD); transient ischaemic attack, subarachnoid haemorrhage, intracerebral haemorrhage, ischaemic stroke, abdominal aortic aneurysm, and peripheral arterial disease (PAD). Secondary endpoints were first event presentation of fatal and non-fatal CVDs (e.g. regardless of the prior occurrence of other type of CVD), composite endpoint CVD (including all cardiovascular endpoints defined in the study except stable angina), and first event of each CVD (i.e. regardless of other earlier CVD presentations). Diagnosis codes used to define each endpoint can be found at http://www.caliberresearch.org/portal/.

### Statistical Analysis

Follow-up of patients was censored on the date of first CVD presentation, death from other causes, last data collection, or deregistration from the practice, whichever happened first. First, the lifetime cumulative incidence of each CVD was estimated using Cox models adjusted for the competing risk of initial presentation with another CVD or death from other causes (or of first presentation with death from other CVD or other causes, as appropriate), and age as the time-scale. In primary analyses, the association between quintiles of small-area socioeconomic deprivation and each endpoint was examined using Cox proportional hazard models stratified by practice. The least deprived quintile was used as the reference category. The proportional hazards assumption was verified by plotting the Schoenfield residuals. The shape of associations across increasing levels of deprivation was further explored using deciles of index of multiple deprivation. Because associations for most CVDs differed by sex (*P*-value of likelihood ratio for interaction <0.05), analyses are presented separately for men and women. Models were initially adjusted for age only, and then further adjusted for cardiovascular factors. Missing covariate data were imputed using multiple imputation (Text S2). Assuming independence in effects, heterogeneity in associations across CVD endpoints for the fifth vs. the first quintile was assessed with τ^2^ statistic [38], which is the between-CVD endpoint variance, and I^2^ that can be interpreted as the proportion of the total variation in estimates that is due to heterogeneity. In sensitivity analyses, associations were examined after ignoring endpoints recorded in primary care data, restricting the analyses to fatal events, and including first occurrence of each CVD regardless of other earlier CVD presentation.

In secondary analyses, effect modification by baseline age and calendar period in relation to the introduction of financial reward for performance of medical practices (before/after 1^st^ April 2004) was evaluated. Associations were also assessed within the following patient risk groups measured at baseline: current smokers, hypertensive, obese (body mass index ≥30 kg/m^2^), diabetic, patients with depression, and healthy. Patients not classified in any of the risk groups considered were classified as healthy.

The clinical utility of the IMD to predict the risk of CVD was finally assessed estimating the increment in c-index when information on small-area socioeconomic deprivation was added into a model with age and sex, among patients aged 40–74 years (Vascular Health Screening target group in England). Analyses were performed in Stata12 and R 3.0.

## Results

### Patient characteristics

During the 11.6 million person-years of study follow-up (median 5.5 years per patient) 1,937,360 million individuals accrued 114,859 fatal and non-fatal CVD endpoints. Median age at baseline was 47.2 years and 50.5% were women. Proportions of people who were non-white, current smokers, obese, diabetic, and diagnosed with depression were higher in socioeconomically deprived quintile groups (Table 1). Consultation rates were also higher amongst the most socially deprived individuals and were consistently higher in women than in men.

**Table 1:** Baseline patient characteristics by quintile of small-area socioeconomic deprivation in men and women.

	Overall	Men					Women				
		Q1 (Least)	Q2	Q3	Q4	Q5(Most)	Q1(Least)	Q2	Q3	Q4	Q5 (Most)
**Number of patients**	1,937,360	191,608	190,112	189,029	192,049	195,531	197,553	197,846	196,668	195,710	191,254
**Mean age, years [SD]**	47.2 [15.4]	46.2 [13.8]	46.6 [14.4]	46.5 [14.4]	45.4 [14.3]	44.4 [14.1]	48.5 [15.8]	49.3 [16.3]	49.2 [16.4]	48.4 [16.6]	47.3 [16.5]
**Ethnicity** * **(%)**											
White	90.5	95.1	95.2	91.0	89.8	82.6	94.7	95.0	91.4	90.0	83.3
South Asian	2.9	1.9	1.7	3.7	3.3	4.5	1.9	1.6	3.2	2.9	4.0
Black	3.1	0.8	0.9	1.7	2.8	8.2	0.9	0.9	1.7	3.0	8.2
**Smoking status (%)**											
Current	20.3	15.5	19.4	21.5	27.3	35.6	11.0	14.1	15.9	20.6	27.1
Ex-smoker	16.2	18.3	18.9	18.6	18.0	16.1	14.8	15.1	14.7	15.1	13.7
Never smoker	63.5	66.2	61.7	59.9	54.8	48.3	74.2	70.8	69.4	64.4	59.2
**Hypertension** * **(%)**	53.3	56.7	59.5	58.9	57.2	55.6	47.4	50.9	51.6	51.2	51.8
**Blood pressure lowering medication (%)**	16.3	12.3	13.1	13.1	12.5	12.5	18.5	19.8	19.9	20.1	21.1
**Mean SBP** * **, mmHg [SD]**	130 [19.0]	133 [16.9]	134 [17.2]	133 [17.2]	133 [17.4]	132 [17.6]	127 [19.3]	128 [19.7]	128 [19.9]	128 [20.0]	128 [20.1]
**Mean DBP** * **, mmHg [SD]**	78 [10.3]	80 [9.8]	80 [9.8]	80 [10.0]	80 [10.1]	80 [10.3]	77 [10.1]	77 [10.2]	77 [10.2]	77 [10.3]	77 [10.5]
**Diabetes mellitus (%)**	2.6	2.3	2.6	2.9	3.0	3.2	1.7	2.0	2.4	2.7	3.3
**Obesity** * **(%)**	20.0	16.8	18.0	19.2	20.5	20.9	15.4	18.1	19.9	22.8	27.4
**Mean BMI** * **, kg/m^2^ [SD]**	26.4 [5.2]	26.6 [4.1]	26.7 [4.3]	26.8 [4.5]	26.8 [4.7]	26.7 [5.0]	25.3 [5.1]	25.8 [5.4]	26.0 [5.6]	26.5 [5.9]	27.2 [6.4]
**Mean total cholesterol, mmol/L [SD]**	5.4 [1.2]	5.3 [1.1]	5.3 [1.1]	5.3 [1.2]	5.3 [1.1]	5.2 [1.2]	5.5 [1.1]	5.5 [1.2]	5.5 [1.1]	5.5 [1.2]	5.4 [1.2]
**Mean HDL cholesterol, mmol/L [SD]**	1.4 [0.4]	1.3 [0.4]	1.3 [0.4]	1.3 [0.4]	1.2 [0.4]	1.2 [0.4]	1.6 [0.5]	1.6 [0.5]	1.6 [0.4]	1.5 [0.4]	1.5 [0.4]
**Statin use (%)**	2.9	2.8	3.1	3.3	3.0	3.0	2.2	2.6	2.8	2.8	3.2
**Depression**	2.3	1.4	1.6	1.6	1.8	2.1	2.6	2.7	2.7	3.1	3.2
**Mean consultation rate [SD]**	5.6 [6.6]	3.9 [5.1]	4.1 [5.4]	4.2 [5.7]	4.4 [6.1]	4.8 [6.6]	6.5 [6.5]	6.7 [6.9]	6.8 [7.0]	6.9 [7.3]	7.5 [7.8]

Note: BMI, body mass index; DBP, diastolic blood pressure; HDL, high density lipoprotein; SD; standard deviation; SBP, systolic blood pressure; Q, quintile of social deprivation.

*Missing values for ethnicity = 47.4%; hypertension = 50.2%; obesity and BMI = 69.5%; SBP and DBP = 57.5%.

### Lifetime cumulative incidence of 12 presentations of CVD

**Figure 1:**
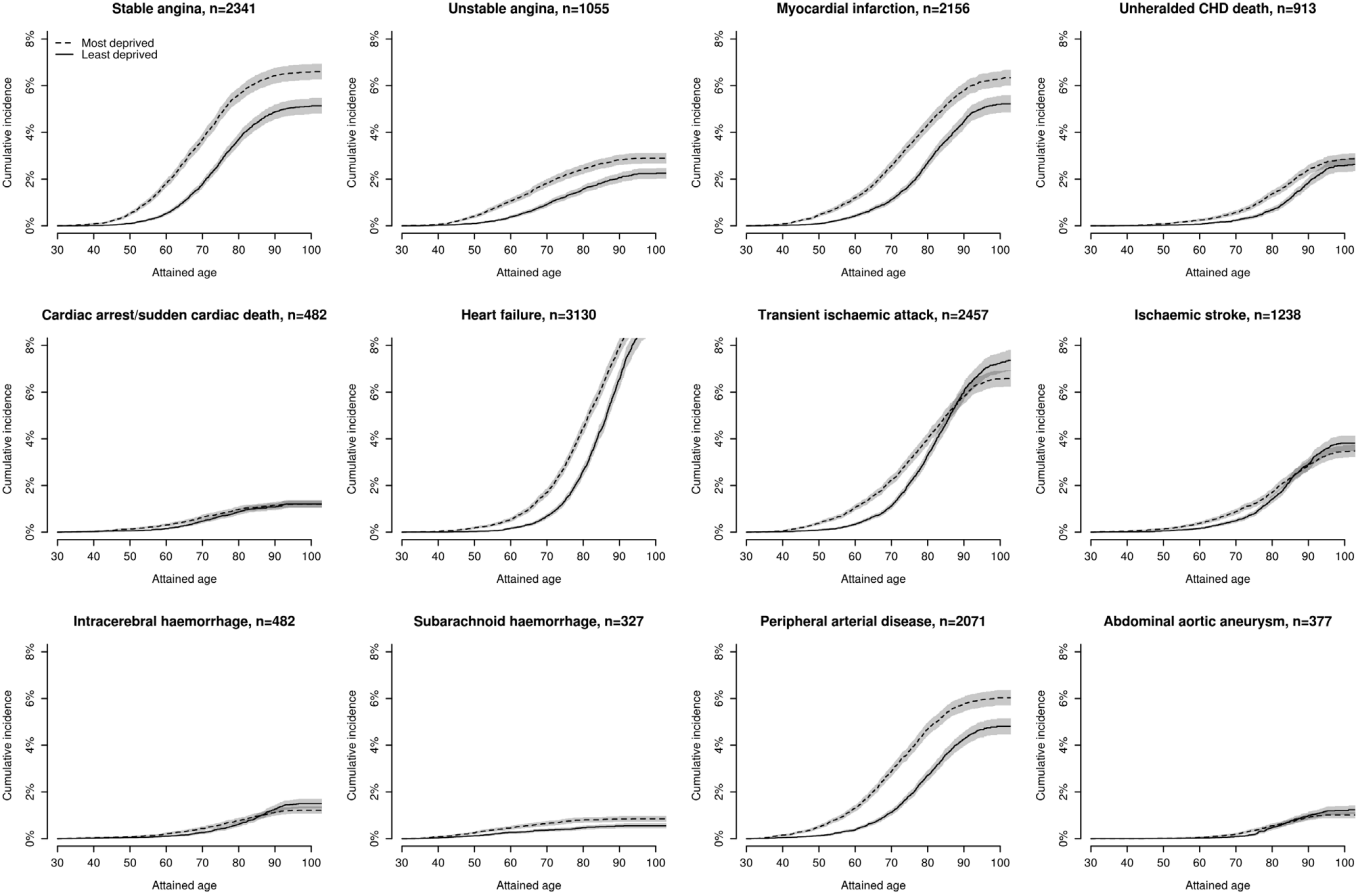
Lifetime cumulative incidence of 12 cardiovascular diseases stratified by quintiles of socioeconomic deprivation in women.

**Figure 2:**
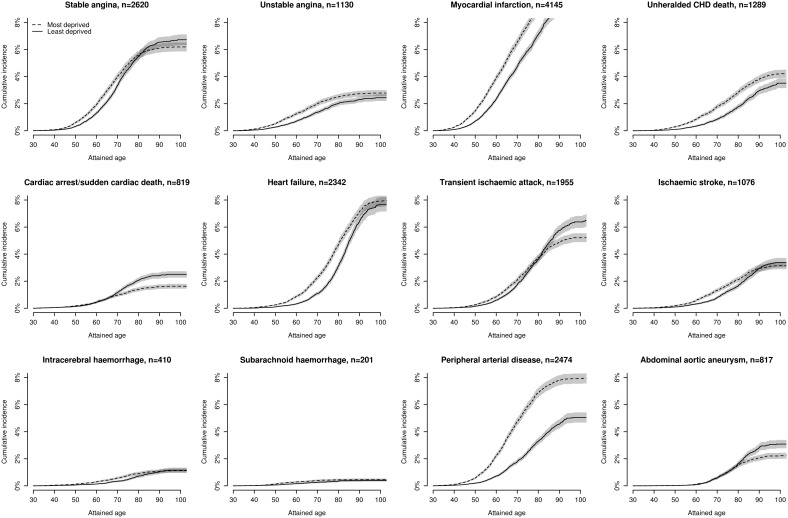
Lifetime cumulative incidence of 12 cardiovascular diseases stratified by quintiles of socioeconomic deprivation in men.

Small-area deprivation had little effect on the cumulative lifetime incidence of many initial CVD presentations in women (Figure 1 and Table S1) and still less effect in men (Figure 2 and Table S2). Thus, in both sexes, initial presentations with CA-SCD, transient ischaemic attack, abdominal aortic aneurysm and ischaemic and intracerebral haemorrhage were no more frequent in the most deprived than least deprived quintile groups. In men, initial presentation with stable angina was also unaffected by small-area deprivation. Other CVD presentations showed a graded increase in cumulative lifetime incidence with worsening small-area deprivation, the largest increase by age 90 years being for peripheral arterial disease in women (4.3% in the least deprived, 5.8% in the most deprived) and men (4.6% in least deprived, 7.8% in the most deprived quintile groups).

### Small-area socioeconomic deprivation and 12 presentations of CVD

**Figure 3:**
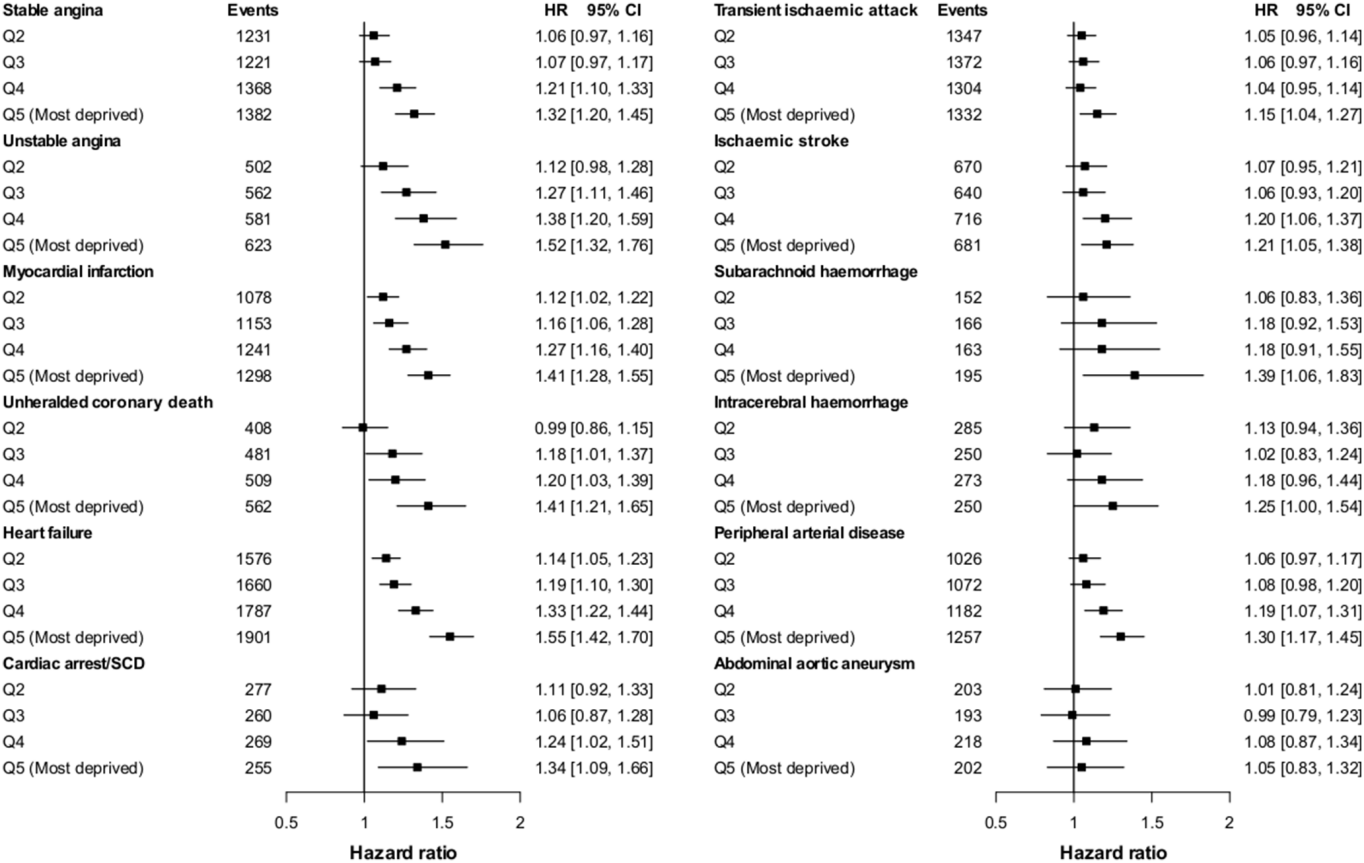
Hazard ratios for the association between the initial presentation of 12 cardiovascular diseases and socioeconomic deprivation (ref. least deprived quintile) adjusted for common cardiovascular risk factors measured at baseline in women.

**Figure 4:**
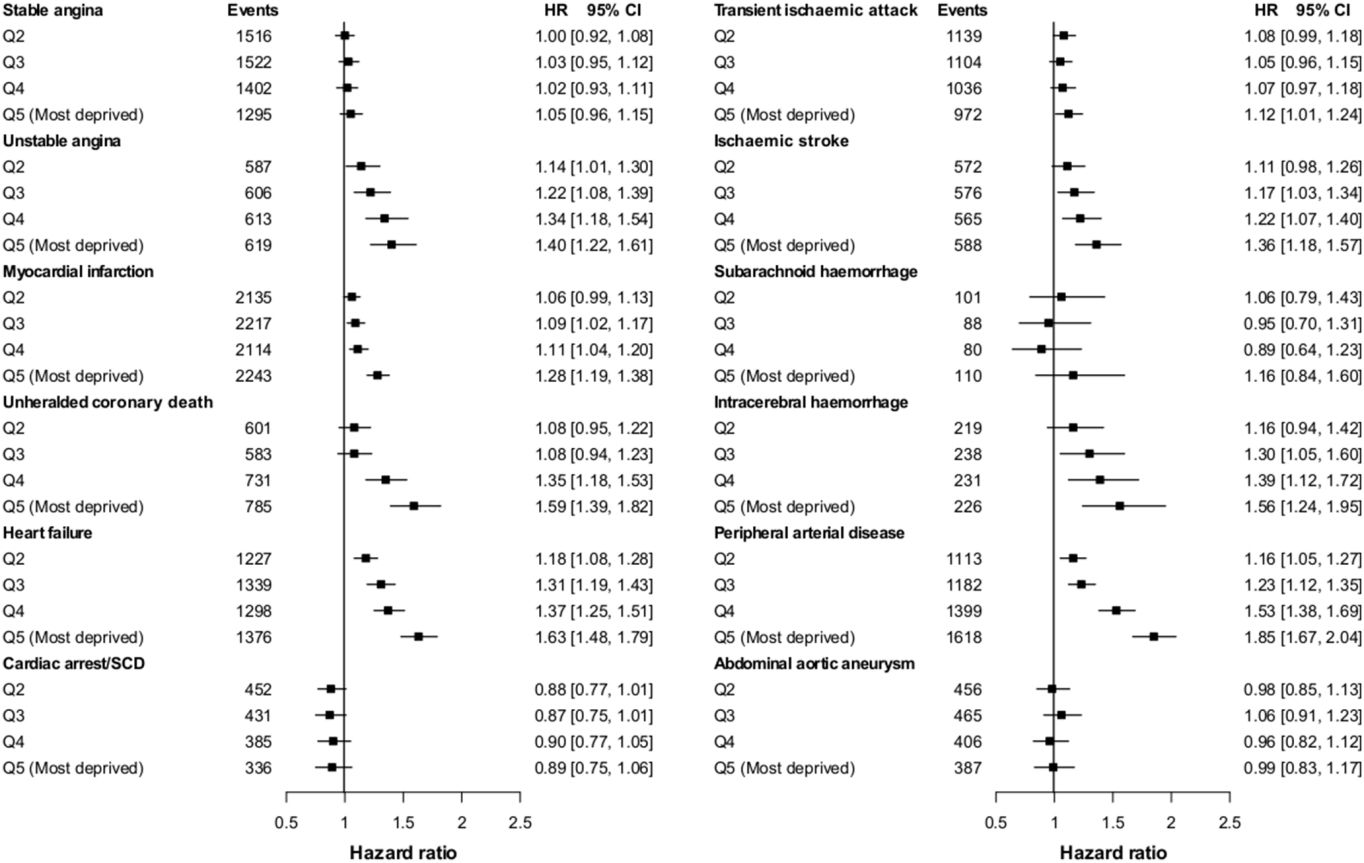
Hazard ratios for the association between the initial presentation of 12 cardiovascular diseases and socioeconomic deprivation (ref. least deprived quintile) adjusted for common cardiovascular risk factors measured at baseline in men.

Analysis of hazard ratios for initial presentations of CVDs confirmed considerable heterogeneity in both women (I^2^ = 81.9%, τ^2^ = 0.01) and men (I^2^ = 96.0%, τ^2^ = 0.06). In women (Figure 3), small-area socioeconomic deprivation showed no association with abdominal aortic aneurysm. In men too there was no association with abdominal aortic aneurysm and no association with stable angina, CA-SCD, subarachnoid haemorrhage or transient ischemic attack (Figure 4). For other CVDs hazard increased linearly with deprivation quintile, the increase being comparable between the sexes for unstable angina, myocardial infarction and heart failure, but steeper in men for peripheral arterial disease, ischemic stroke and intracerebral haemorrhage (from adjusted HR = 1.16 to 1.85; HR = 1.11 to 1.36; and HR = 1.16 to 1.56; respectively, for second least deprived and most deprived quintiles vs. least deprived) compared with women (from adjusted HR = 1.06 to 1.30; HR = 1.07 to 1.21; and HR = 1.13 to 1.25; respectively). Results were robust to sensitivity analyses excluding primary care endpoint data or non-fatal events, or including first occurrence of CVD regardless of other prior CVD presentation (Figures S14 & S15). Further adjustment for ethnicity (n = 1,018,538) did not change the estimates (Figures S10 & S11).

### Interactions with age and pay for performance period

The relationship between small-area socioeconomic deprivation and initial presentations with CVD weakened with older age, disappearing completely by the age of 70 years for all presentations except peripheral arterial disease in men and unheralded coronary death and heart failure in both men and women (Figures S4 & S5). No difference in associations between small-area socioeconomic deprivation and each of the 12 CVDs was seen before or after the introduction of the pay for performance policy (Figures S8 & S9).

### Associations in high risk groups

The hazard of different CVDs in its associations with small-area socioeconomic deprivation showed little evidence of attenuation in high risk men and women. Thus in subgroups with hypertension, obesity, diabetes, depression and current smoking the hazard ratio of quintile 5 vs. quintile 1 deprivation remained remarkably constant and comparable to the hazard ratios in a healthy subgroup without these risk factors. (Figures S6 & S7).

### Discriminative ability of multiple deprivation index

**Figure 5:**
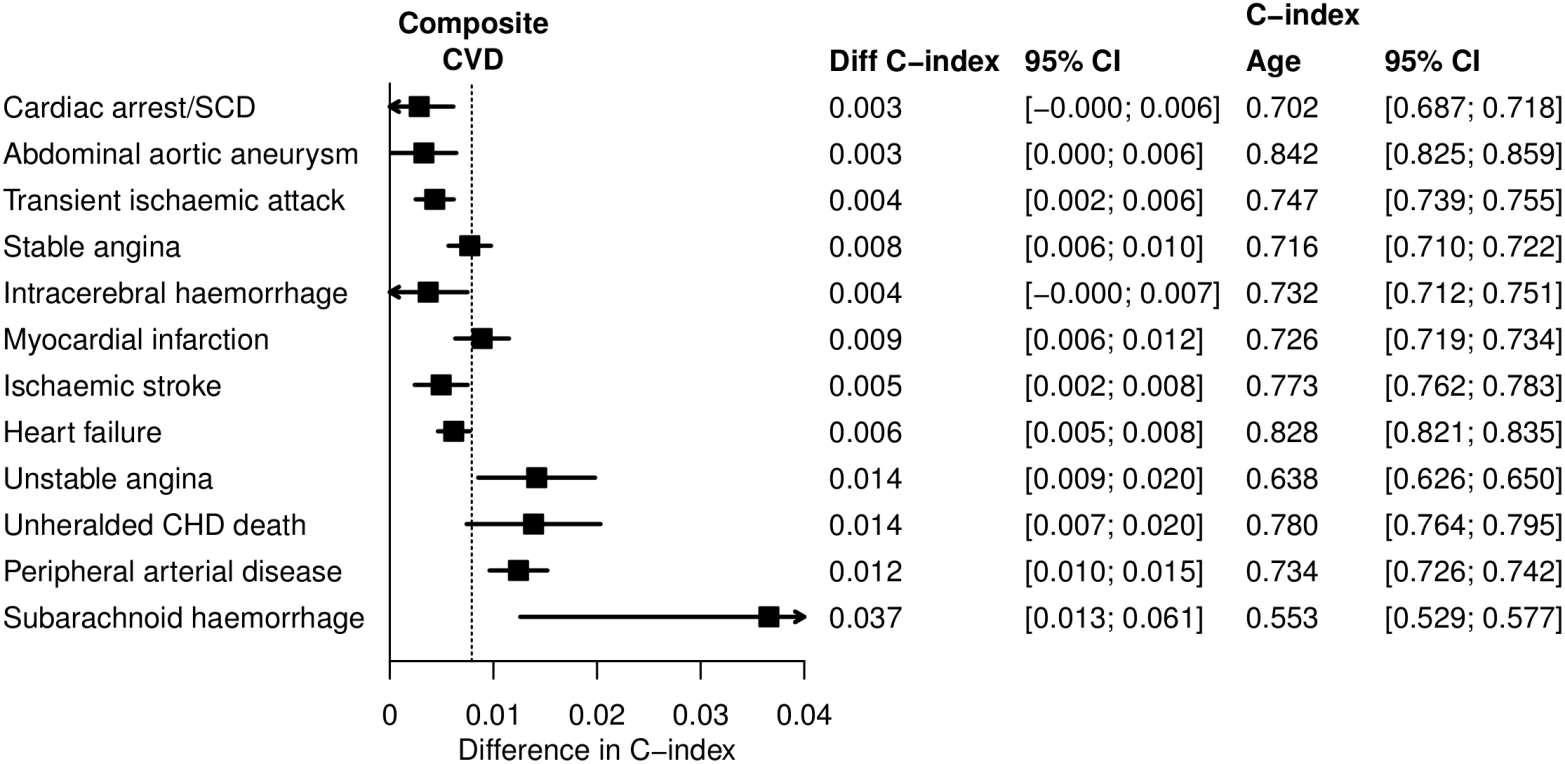
Increment in c-index associated with inclusion of socioeconomic deprivation in cardiovascular phenotype specific models containing age in women.

**Figure 6:**
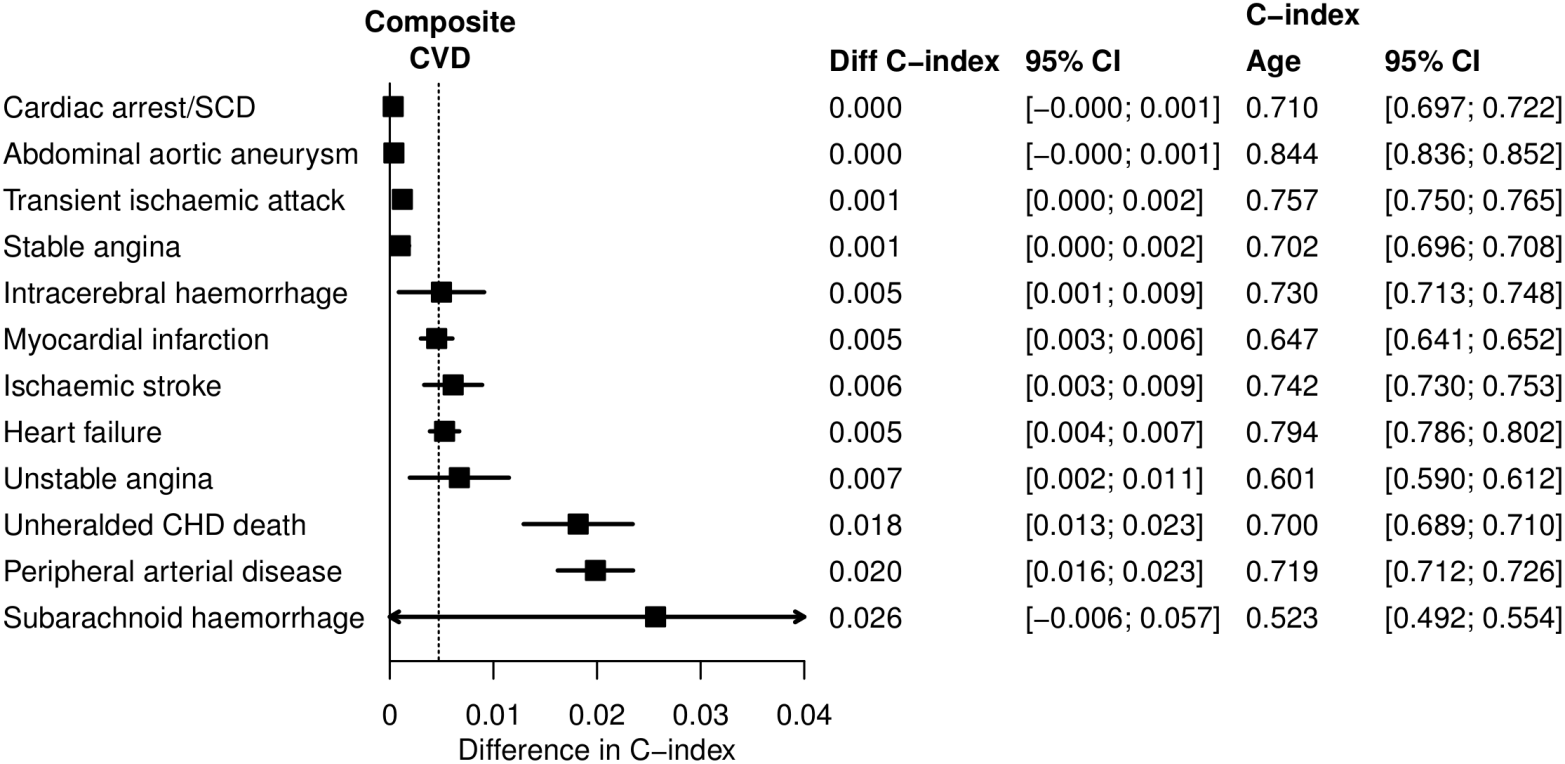
Increment in c-index associated with inclusion of socioeconomic deprivation in cardiovascular phenotype specific models containing age in men.

Increments in c-index of 0.8% (95%CI 0.7–0.9) for women and 0.5% (95%CI 0.4–0.5) for men, represented by the vertical lines in figures 5 and 6, quantify the enhanced discrimination when small-area socioeconomic deprivation is included in age adjusted risk prediction models for a composite of all 12 CVD presentations. However, for individual CVD presentations the increments in c-index were variable and in men, negligible for CA-SCD, abdominal aortic aneurysm, transient ischaemic attack and stable angina, more substantial for unheralded coronary death and peripheral arterial disease. In women variation was less marked, and for each of the 12 CVD presentations increments in c-index were observed, particularly for unstable angina, myocardial infarction, unheralded coronary death, peripheral arterial disease and subarachnoid haemorrhage.

### Ethical considerations

The analysis was approved by the Independent Scientific Advisory Committee of the Medicines and Healthcare products Regulatory Agency and the Myocardial Ischaemia National Audit Project Academic Group. The protocol was registered at clinicaltrials.gov (NCT01937065). Data from patients attending the general (primary care) practices consenting to data linkage for CALIBER were included in the analysis.

## Discussion

In a contemporary population-based cohort of nearly 2 million women and men, associations of small-area socioeconomic deprivation with 12 different presentations of CVD were heterogeneous and often absent, particularly in men, with only small effects on the lifetime cumulative incidence of disease. In both sexes we confirmed graded associations of small-area deprivation with myocardial infarction and coronary death but found no association with abdominal aortic aneurysm. Men, unlike women, also showed no association with stable angina, sudden cardiac death, subarachnoid haemorrhage or transient ischaemic attack. Associations were similar in subgroups with and without cardiovascular risk factors and did not change after introduction of the pay for performance policy.

Previous reports of deprivation and its association with CVD have focused on myocardial infarction, and coronary mortality [6], [7], [9], [14]. However, these outcomes account for less than a third of cardiovascular presentations [28] and provide, therefore, an incomplete picture of how deprivation affects disease development. Only limited information is available about other manifestations of CVD, two recent studies reporting increased risk of heart failure with higher deprivation level [39], [40], and one reporting increased risk of peripheral arterial disease [21]. We have addressed the limitations of previous studies by using linked electronic health records [28] to assemble the largest cohort yet studied, allowing us to examine associations of small-area deprivation status with the hazard of initial presentation and cumulative incidence of a broad range of CVDs in both women and men.

We have shown for the first time that assumptions based on the widely reported association of socioeconomic deprivation with myocardial infarction, and coronary mortality cannot be generalized to other manifestations of CVD. This is particularly true for men in whom small-area socioeconomic deprivation had no effect on the hazard of presenting with over one third of the CVDs analysed. In contrast, we found consistently graded associations in women, the hazard of presenting with abdominal aortic aneurysm being the only exception in showing no association with socioeconomic deprivation. Despite the reported associations between small-area deprivation and some CVD presentations in men and most CVD presentations in women, analysis of cumulative incidence showed that across a lifetime the effects were often negligible, incidence rates by deprivation quintile being almost identical in about a half of CVD presentations in women and men. Only for peripheral arterial disease, heart failure, myocardial infarction and coronary death was the fifth quintile of deprivation associated with more substantial increases in the lifetime cumulative incidence of disease. These findings are consistent with those reported in the literature for specific CVDs including myocardial infarction [12], [14], heart failure [40]–[42], peripheral arterial disease [20], and cardiac arrest [18].

The heterogeneous associations of small-area deprivation by gender and by CVD phenotype emphasise the importance of developing separate risk models for women and men and caution against those based on composite cardiovascular endpoints. Thus we found that inclusion of small-area deprivation coefficients in risk prediction models had variable, sometimes large, effects on the discriminative function depending on gender and on the type of CVD. Age was an important modifier of deprivation effects, hazard ratios for most CVD presentations diminishing in older women and men. Only presentation with peripheral arterial disease in men and unheralded coronary death and heart failure in both men and women retained an association with deprivation beyond the age of 70 years. It is salutary that the cardiovascular health risk attributable to small-area deprivation falls most heavily on younger people, particularly women, identifying them as a priority target for policies aimed at reducing socioeconomic division.

Our data do not permit robust mechanistic explanations for the heterogeneity observed in this study, which likely reflects differences in disease biology, stable angina, for example, resulting from atheromatous coronary stenosis, acute coronary syndromes from thrombotic coronary occlusion and haemorrhagic stroke from vascular rupture. Also potentially important are sex differences in disease biology, women, for example, being less susceptible to acute myocardial infarction but similarly susceptible to stable coronary disease [43], [44]. It is quite plausible that a complex risk factor such as socioeconomic deprivation might have very different effects on the pathophysiology of these biologically diverse clinical presentations and that these effects might differ between women and men. Other factors that merit consideration are the differential exposure to conventional risk factors across deprivation groups that we, like other investigators, have reported [6], [45], and the differential susceptibility of women and men to disease development in response to exposure [46], [47]. Overall, our findings suggest increased susceptibility to the adverse effects of deprivation in women and support the use of small-area socioeconomic deprivation in developing gender-based risk models and screening tools [8], [27].

In agreement with previous reports [48], [49], we found no evidence that the increased risk of a range of CVD presentations associated with higher deprivation declined after the introduction of the Quality and Outcomes Framework in 2004, a scheme incentivising United Kingdom primary care physicians to apply management strategies to reduce the risk of CVD. This together with the finding of similarity of hazards between high risk groups and healthy patients suggests a failure of current preventive strategies to mitigate health inequalities, probably reflecting the complex interactions between small-area deprivation and CVD.

A number of limitations require consideration when interpreting the results of this study. First, the index of multiple deprivation provide information on living circumstances which are not captured by individual-level information. Previous research has shown that social position and area deprivation are associated but that there is substantial variability in the characteristics of the area of residence within categories of social class [50], education or income [51]. Because social class data was unavailable in the CALIBER dataset, it was not possible to estimate the extent of overlap between area and individual level measures of deprivation. However, previous studies have shown that the association between area measures of deprivation and coronary heart disease is independent from measures of socioeconomic position such as occupation, education or income [23]. And a recent randomised experiment provided evidence of physical and mental health improvement following an intervention which changed the area of residence of individuals [1], [2]. Second, information about certain health behaviours, such as diet, heavy alcohol use or physical activity, and about individual socioeconomic status was not accounted for in adjustment [52] and residual confounding cannot be excluded. We were also unable to examine the potential modifier effect of individual social position of people living in different deprivation areas. In addition, covariate data were missing for a number of individuals but adjusted estimates based on imputed data were consistent with those obtained in age adjusted models in men and women (Figures S2 & S3). Third, despite of the large sample size, the study power for some of the secondary analyses such as the assessment of differences in the periods before and after the implementation of the pay for performance policy, or association in the subgroup of patients with depression was limited, especially for the more rare cardiovascular endpoints (i.e.CA-SCD, subarachnoid haemorrhage and intracerebral haemorrhage). Finally, CVDs were defined using data from four different data sources, each of which has its own error. However, associations with small-area deprivation were robust to exclusion of primary care cases or non-fatal cases; and we [53] and others [54] have provided evidence of the validity of using linkages for endpoint follow-up.

### Conclusion

The conventional view of socioeconomic deprivation as a risk factor for CVD is based largely on studies of acute myocardial infarction and coronary death and takes little account of the wider range of CVD presentations or of differences between women and men. By deconstructing CVD into its clinically diverse presentations, we have shown that associations with small-area deprivation are heterogeneous and while women exhibit a graded increase in risk across deprivation quintiles, associations are less marked and often absent in men. In both sexes the contribution that small-area deprivation makes to lifetime risk is often small and for some presentations non-existent. These findings suggest that policies to reduce deprivation will impact more strongly on CVD incidence in women than men, with greater effects on angina, myocardial infarction and heart failure than abdominal aortic aneurysm.

## Supporting Information

Figure S1 **Study flow diagram.**
(TIFF)

Figure S2 **Age adjusted hazard ratios for the association between the initial presentation of twelve cardiovascular diseases and socioeconomic deprivation (ref. least deprived quintile) in women.** Note: CI, confidence interval; HR, hazard ratios; Q, quintile; SCD, sudden cardiac death; *, p-value of likelihood ratio test for trend <0.05. P-values of likelihood ratio test for interaction with sex were <0.0001 for stable angina and PAD, 0.003 for MI and 0.004 for CA-SCD.
(TIF)

Figure S3 **Age adjusted hazard ratios for the association between the initial presentation of twelve cardiovascular diseases and socioeconomic deprivation (ref. least deprived quintile) in men.** Note: CI, confidence interval; HR, hazard ratios; Q, quintile; SCD, sudden cardiac death; *, p-value of likelihood ratio test for trend <0.05. P-values of likelihood ratio test for interaction with sex were <0.0001 for stable angina and PAD, 0.003 for MI and 0.004 for CA-SCD.
(TIF)

Figure S4 **Hazard ratios for the association between the initial presentation of twelve cardiovascular diseases per quintile increase in socioeconomic deprivation by age group in women.** Note: CI, confidence interval; HR, hazard ratios adjusted for smoking status, systolic blood pressure, total and high-density lipoprotein cholesterol and body mass index; Q, quintile; SCD, sudden cardiac death; *, p-value of likelihood ratio test for interaction <0.001; **, p-value of likelihood ratio test for interaction <0.01.
(TIF)

Figure S5 **Hazard ratios for the association between the initial presentation of twelve cardiovascular diseases per quintile increase in socioeconomic deprivation by age group in men.** Note: CI, confidence interval; HR, hazard ratios adjusted for smoking status, systolic blood pressure, total and high-density lipoprotein cholesterol and body mass index; Q, quintile; SCD, sudden cardiac death; *, p-value of likelihood ratio test for interaction <0.01; **, p-value of likelihood ration for interaction ≤0.05.
(TIF)

Figure S6 **Hazard ratios for the association between most vs. least quintile of socioeconomic deprivation and twelve cardiovascular diseases in healthy individuals, current smokers, patients with hypertension, obesity, diabetes or depression in women.** Note: CI, confidence interval; Healthy, patients without hypertension, obesity, diabetes or depression and not currently smoking; HR, hazard ratios; SCD, sudden cardiac death.
(TIF)

Figure S7 **Hazard ratios for the association between most vs. least quintile of socioeconomic deprivation and twelve cardiovascular diseases in healthy individuals, current smokers, patients with hypertension, obesity, diabetes or depression in men.** Note: CI, confidence interval; Healthy, patients without hypertension, obesity, diabetes or depression and not currently smoking; HR, hazard ratios; SCD, sudden cardiac death.
(TIF)

Figure S8 **Age adjusted hazard ratios for the association between most vs. least quintile of socioeconomic deprivation and twelve cardiovascular diseases in the periods before and after the introduction of pay for performance (April 2004) in women.** Note: CI, confidence interval; HR, hazard ratios adjusted for smoking status, systolic blood pressure, total and high-density lipoprotein cholesterol and body mass index; SCD, sudden cardiac death.
(TIF)

Figure S9 **Age adjusted hazard ratios for the association between most vs. least quintile of socioeconomic deprivation and twelve cardiovascular diseases in the periods before and after the introduction of pay for performance (April 2004) in men.** Note: CI, confidence interval; HR, hazard ratios adjusted for smoking status, systolic blood pressure, total and high-density lipoprotein cholesterol and body mass index; SCD, sudden cardiac death.
(TIF)

Figure S10 **Hazard ratios for the association between the initial presentation of twelve cardiovascular diseases and socioeconomic deprivation (ref. least deprived quintile) adjusted for ethnicity and for common cardiovascular risk factors measured at baseline in women.** Note: CI, confidence interval; HR, hazard ratios adjusted for age, ethnicity, smoking status, diabetes type, systolic blood pressure, total and high-density lipoprotein cholesterol and body mass index; Q, quintile; SCD, sudden cardiac death.
(TIF)

Figure S11 **Hazard ratios for the association between the initial presentation of twelve cardiovascular diseases and socioeconomic deprivation (ref. least deprived quintile) adjusted for ethnicity and for common cardiovascular risk factors measured at baseline in men.** Note: CI, confidence interval; HR, hazard ratios adjusted for age, ethnicity, smoking status, diabetes type, systolic blood pressure, total and high-density lipoprotein cholesterol and body mass index; Q, quintile; SCD, sudden cardiac death.
(TIF)

Figure S12 **Age adjusted hazard ratios for the association between the initial presentation of twelve cardiovascular diseases and socioeconomic deprivation (ref. least deprived quintile) by ethnic group.** Note: CI, confidence interval; HR, hazard ratios; Q, quintile; SCD, sudden cardiac death.
(TIF)

Figure S13 **Adjusted hazard ratios for the association between most vs. least quintile of socioeconomic deprivation and twelve cardiovascular diseases by source of endpoint.** Note: CI, confidence interval; HR, hazard ratios adjusted for age, ethnicity, smoking status, diabetes type, systolic blood pressure, total and high-density lipoprotein cholesterol and body mass index; SCD, sudden cardiac death.
(TIF)

Figure S14 **Adjusted hazard ratios for the association between most vs. least quintile of socioeconomic deprivation and twelve initial cardiovascular disease presentations vs. first event presentations in women.** Note: CI, confidence interval; HR, hazard ratios adjusted for age, ethnicity, smoking status, diabetes type, systolic blood pressure, total and high-density lipoprotein cholesterol and body mass index; Q, quintile; SCD, sudden cardiac death.
(TIF)

Figure S15 **Adjusted hazard ratios for the association between most vs. least quintile of socioeconomic deprivation and twelve initial cardiovascular disease presentations vs. first event presentations in men.** Note: CI, confidence interval; HR, hazard ratios adjusted for age, ethnicity, smoking status, diabetes type, systolic blood pressure, total and high-density lipoprotein cholesterol and body mass index; Q, quintile; SCD, sudden cardiac death.
(TIF)

Table S1 **Lifetime risks of initial vs. first event presentation of twelve cardiovascular diseases by level of socioeconomic deprivation for consecutive attained ages in women.** Note: CA-SCD, atrial fibrillation, cardiac arrest and sudden cardiac death; CI, confidence interval; first event, first cardiovascular disease presentation of a specific type, regardless of prior occurrence of another type of cardiovascular disease; initial presentation, first presentation of cardiovascular disease of any type for a specific patient.
(DOCX)

Table S2 **Lifetime risks of initial vs. first event presentation of twelve cardiovascular diseases by level of socioeconomic deprivation for consecutive attained ages in omen.** Note: CA-SCD, atrial fibrillation, cardiac arrest and sudden cardiac death; CI, confidence interval; first event, first cardiovascular disease presentation of a specific type, regardless of prior occurrence of another type of cardiovascular disease; initial presentation, first presentation of cardiovascular disease of any type for a specific patient.
(DOCX)

Text S1 **CALIBER program: study data sources.**
(DOCX)

Text S2 **Multiple imputation.**
(DOCX)
