# Supplementary figures and images for: Socioeconomic Deprivation and the Incidence of 12 Cardiovascular Diseases in 1.9 Million Women and Men: Implications for Risk Prediction and Prevention
Source: PLoS One. 2014 Aug 21;9(8):e104671. doi: 10.1371/journal.pone.0104671 (PMC4140710; doi:10.1371/journal.pone.0104671)

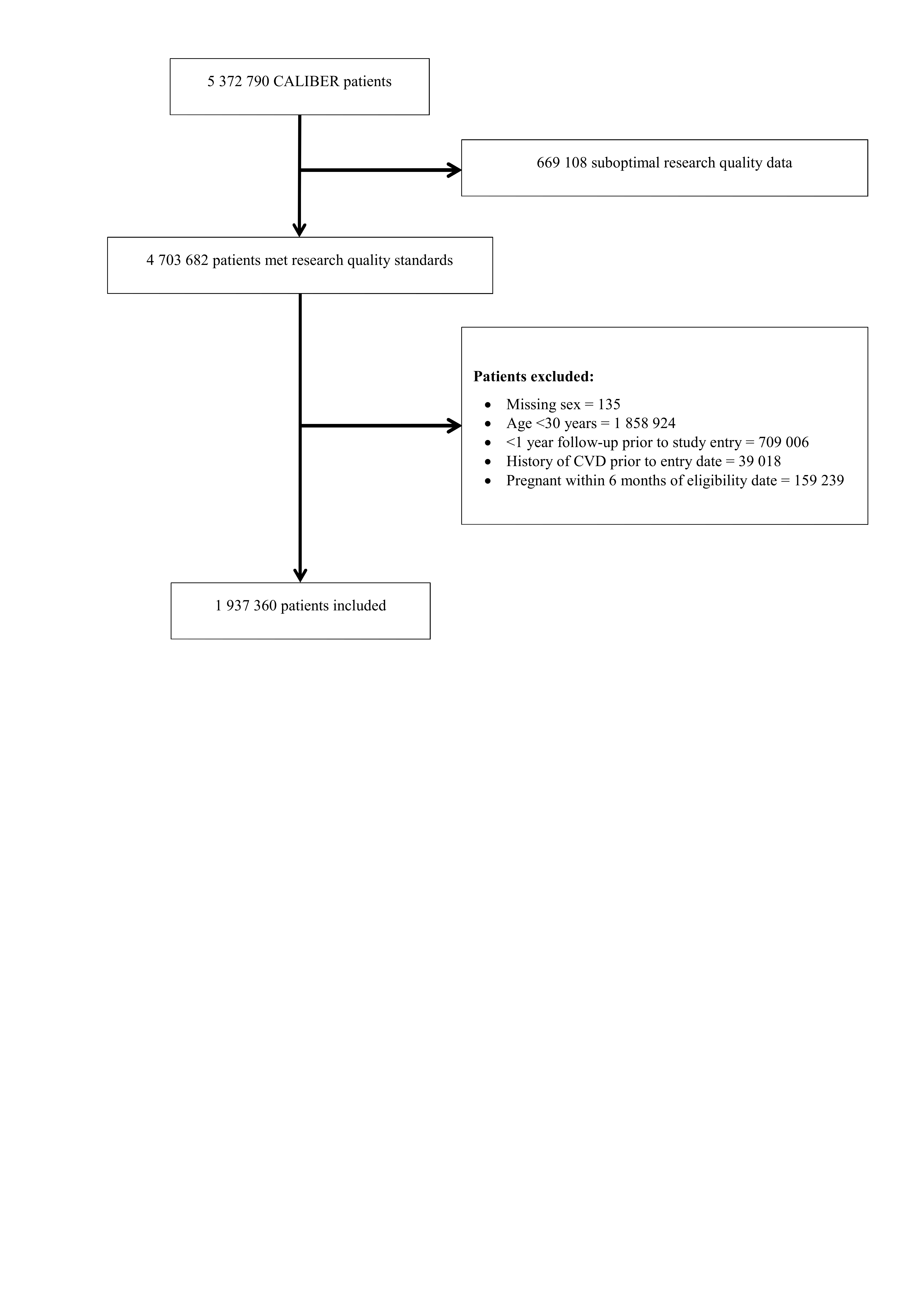

Supplement: Figure S1 — Study flow diagram. (TIFF) [file pone.0104671.s001.tiff]
