# Supplementary material for: Socioeconomic Deprivation and the Incidence of 12 Cardiovascular Diseases in 1.9 Million Women and Men: Implications for Risk Prediction and Prevention
Source: PLoS One. 2014 Aug 21;9(8):e104671. doi: 10.1371/journal.pone.0104671 (PMC4140710; doi:10.1371/journal.pone.0104671)
Supplement: Table S1 — Lifetime risks of initial vs. first event presentation of twelve cardiovascular diseases by level of socioeconomic deprivation for consecutive attained ages in women. Note: CA-SCD, atrial fibrillation, cardiac arrest and sudden cardiac death; CI, confidence interval; first event, first cardiovascular disease presentation of a specific type, regardless of prior occurrence of another type of cardiovascular disease; initial presentation, first presentation of cardiovascular disease of any type for a specific patient. (DOCX) [file pone.0104671.s016.docx]

**Table S2-1-1 Lifetime risk of initial vs. first event presentation of twelve cardiovascular diseases by level of socioeconomic deprivation for consecutive attained ages in women**

| **Age (years)** | **Least deprived (Q1)** | | **Q3** | | | **Most deprived (Q5)** | |  |
| --- | --- | --- | --- | --- | --- | --- | --- | --- |
|  | **Percentage lifetime risk (95% CI)** | | **Percentage lifetime risk (95% CI)** | | | **Percentage lifetime risk (95% CI)** | |  |
|  | **Initial presentation** | **First event** | **Initial presentation** | | **First event** | **Initial presentation** | **First event** |  |
| **Stable angina** |  |  |  | |  |  |  |  |
| 40 | 0.01 (0-0.03) | 0.04 (0.02-0.07) | 0.02 (0-0.04) | | 0.06 (0.03-0.08) | 0.09 (0.05-0.12) | 0.18 (0.13-0.22) |  |
| 50 | 0.10 (0.06-0.13) | 0.24 (0.18-0.291) | 0.21 (0.15-0.26) | | 0.45 (0.37-0.53) | 0.53 (0.44-0.61) | 1.19 (1.06-1.32) |  |
| 60 | 0.53 (0.45-0.61) | 1.21 (1.09-1.34) | 0.95 (0.83-1.06) | | 1.97 (1.80-2.13) | 1.82 (1.65-1.99) | 3.89 (3.64-4.15) |  |
| 70 | 0.79 (0.63-1.96) | 3.67 (3.42-3.91) | 2.47 (2.27-2.66) | | 5.04 (4.76-5.31) | 3.72 (3.46-3.97) | 8.31 (7.92-8.70) |  |
| 80 | 3.71 (3.45-3.97) | 8.11 (7.71-8.52) | 4.38 (4.11-4.64) | | 9.88 (9.47-10.30) | 5.60 (5.29-5.91) | 13.79 (13.28-14.30) |  |
| 90 | 4.88 (4.56-5.20) | 11.66 (11.11-12.21) | 5.42 (5.11-5.72) | | 13.32 (12.79-13.84) | 6.43 (6.09-6.76) | 16.98 (16.39-17.57) |  |
| **Unstable angina** |  |  |  | |  |  |  |  |
| 40 | 0.02 (0.01-0.04) | 0.04 (0.02-0.06) | 0.02 (0-0.03) | | 0.03 (0.01-0.05) | 0.06 (0.03-0.09) | 0.10 (0.06-0.13) |  |
| 50 | 0.10 (0.06-0.13) | 0.16 (0.12-0.21) | 0.22 (0.16-0.27) | | 0.32 (0.25-0.38) | 0.40 (0.33-0.48) | 0.65 (0.55-0.75) |  |
| 60 | 0.39 (0.32-0.46) | 0.62 (0.53-0.70) | 0.58 (0.50-0.67) | | 0.98 (0.87-1.10) | 1.06 (0.93-1.19) | 1.90 (1.73-2.08) |  |
| 70 | 0.92 (0.80-1.04) | 1.66 (1.50-1.83) | 1.28 (1.15-1.42) | | 2.27 (2.08-2.45) | 1.83 (1.65-2.01) | 3.62 (3.36-3.88) |  |
| 80 | 1.57 (1.41-1.74) | 3.40 (3.14-3.67) | 1.98 (1.80-2.16) | | 4.12 (3.85-4.40) | 2.42 (2.21-2.62) | 5.78 (5.44-6.13) |  |
| 90 | 2.06 (1.86-2.27) | 5.33 (4.94-5.73) | 2.38 (2.18-2.58) | | 5.76 (5.40-6.13) | 2.82 (2.60-3.05) | 7.73 (7.30-8.17) |  |
| **Myocardial infarction** |  |  |  | |  |  |  |  |
| 40 | 0.02 (0-0.03) | 0.02 (0-0.03) | 0.03 (0.01-0.05) | | 0.03 (0.01-0.05) | 0.08 (0.05-0.12) | 0.09 (0.06-0.13) |  |
| 50 | 0.09 (0.06-0.12) | 0.09 (0.06-0.13) | 0.19 (0.14-0.24) | | 0.22 (0.16-0.27) | 0.46 (0.38-0.54) | 0.52 (0.44-0.61) |  |
| 60 | 0.43 (0.35-0.50) | 0.49 (0.41-0.57) | 0.68 (0.59-0.78) | | 0.81 (0.70-0.91) | 1.21 (1.07-1.35) | 1.49 (1.34-1.65) |  |
| 70 | 0.12 (0.99-1.25) | 1.40 (1.25-1.55) | 1.62 (1.47-1.78) | | 2.13 (1.94-2.31) | 2.57 (2.36-2.78) | 3.54 (3.27-3.80) |  |
| 80 | 2.72 (2.49-2.94) | 3.86 (3.57-4.15) | 3.44 (3.21-3.68) | | 5.01 (4.70-5.33) | 4.34 (4.06-4.61) | 7.01 (6.62-7.40) |  |
| 90 | 4.46 (4.15-4.78) | 7.71 (7.20-8.22) | 5.20 (4.90-5.51) | | 9.39 (8.88-9.89) | 5.80 (5.49-6.12) | 11.14 (10.60-11.68) |  |
| **Unheralded coronary death** |  |  |  | |  |  |  |  |
| 40 | 0.01 (0-0.02) | 0.01 (0-0.02) | 0 (0-0.01) | | 0 (0-0.01) | 0.01 (0-0.02) | 0.01 (0-0.03) |  |
| 50 | 0.02 (0.01-0.04) | 0.03 (0.01-0.05) | 0.04 (0.02-0.07) | | 0.05 (0.03-0.08) | 0.08 (0.05-0.12) | 0.12 (0.07-0.16) |  |
| 60 | 0.07 (0.04-0.09) | 0.09 (0.05-0.12) | 0.10 (0.06-0.13) | | 0.17 (0.12-0.21) | 0.25 (0.19-0.31) | 0.37 (0.29-0.45) |  |
| 70 | 0.24 (0.17-0.30) | 0.39 (0.31-0.47) | 0.29 (0.22-0.36) | | 0.51 (0.42-0.60) | 0.57 (0.47-0.67) | 0.14 (0.98-1.29) |  |
| 80 | 0.68 (0.56-0.80) | 1.34 (1.16-1.52) | 0.94 (0.81-1.07) | | 2.16 (1.94-2.37) | 1.38 (1.22-1.53) | 3.37 (3.09-3.66) |  |
| 90 | 1.83 (1.61-2.05) | 5.59 (5.10-6.08) | 2.10 (1.89-2.30) | | 6.96 (6.47-7.44) | 2.40 (2.19-2.61) | 8.25 (7.73-8.76) |  |
| **CA-SCD** |  |  |  | |  |  |  |  |
| 40 | 0.03 (0.01-0.05) | 0.03 (0.01-0.05) | 0.03 (0.01-0.06) | | 0.02 (0-0.04) | 0.04 (0.01-0.06) | 0.03 (0.01-0.05) |  |
| 50 | 0.06 (0.03-0.08) | 0.05 (0.03-0.08) | 0.09 (0.05-0.13) | | 0.09 (0.05-0.12) | 0.14 (0.10-0.19) | 0.12 (0.08-0.16) |  |
| 60 | 0.16 (0.11-0.21) | 0.17 (0.12-0.21) | 0.22 (0.17-0.28) | | 0.27 (0.21-0.33) | 0.31 (0.24-0.38) | 0.31 (0.24-0.38) |  |
| 70 | 0.47 (0.39-0.56) | 0.57 (0.48-0.67) | 0.50 (0.41-0.59) | | 0.63 (0.53-0.73) | 0.64 (0.53-0.75) | 0.73 (0.61-0.85) |  |
| 80 | 0.87 (0.74-1.00) | 1.21 (1.05-1.38) | 0.89 (0.77-1.02) | | 1.35 (1.18-1.51) | 0.98 (0.85-1.12) | 1.45 (1.27-1.63) |  |
| 90 | 1.08 (0.93-1.23) | 1.63 (1.42-1.84) | 1.13 (0.98-1.27) | | 1.85 (1.63-2.06) | 1.15 (1.01-1.30) | 1.81 (1.59-2.03) |  |
| **Heart failure** |  |  |  | |  |  |  |  |
| 40 | 0.01 (0-0.02) | 0.01 (0-0.02) | 0.01 (0-0.03) | | 0.02 (0-0.03) | 0.03 (0.01-0.06) | 0.03 (0.01-0.05) |  |
| 50 | 0.03 (0.01-0.05) | 0.04 (0.01-0.06) | 0.06 (0.03-0.08) | | 0.07 (0.04-0.10) | 0.18 (0.13-0.23) | 0.22 (0.16-0.28) |  |
| 60 | 0.17 (0.12-0.22) | 0.20 (0.15-0.26) | 0.24 (0.18-0.30) | | 0.28 (0.22-0.34) | 0.54 (0.45-0.63) | 0.68 (0.57-0.78) |  |
| 70 | 0.70 (0.59-0.81) | 0.85 (0.73-0.98) | 0.86 (0.75-0.98) | | 1.18 (1.04-1.32) | 1.70 (1.52-1.87) | 2.46 (2.23-2.68) |  |
| 80 | 2.65 (2.42-2.88) | 3.79 (3.48-4.09) | 3.18 (2.95-3.42) | | 4.96 (4.64-5.29) | 4.47 (4.19-4.75) | 7.78 (7.36-8.20) |  |
| 90 | 6.56 (6.16-6.96) | 11.38 (10.72-12.04) | 7.12 (6.75-7.49) | | 13.40 (12.77-14.03) | 7.94 (7.57-8.31) | 16.59 (15.91-17.27) |  |
| **Transient ischaemic attack** |  |  |  |  | |  |  | |
| 40 | 0.03 (0.01-0.05) | 0.03 (0.01-0.05) | 0.03 (0.01-0.06) | 0.04 (0.01-0.06) | | 0.05 (0.03-0.08) | 0.06 (0.03-0.09) | |
| 50 | 0.10 (0.06-0.13) | 0.11 (0.08-0.15) | 0.20 (0.15-0.25) | 0.24 (0.19-0.30) | | 0.38 (0.31-0.46) | 0.48 (0.40-0.56) | |
| 60 | 0.34 (0.27-0.40) | 0.40 (0.33-0.47) | 0.59 (0.50-0.68) | 0.76 (0.66-0.86) | | 1.06 (0.93-1.19) | 1.31 (1.16-1.46) | |
| 70 | 1.12 (0.99-1.26) | 1.37 (1.22-1.53) | 1.63 (1.47-1.78) | 2.15 (1.96-2.33) | | 2.24 (2.04-2.44) | 3.08 (2.84-3.33) | |
| 80 | 3.31 (3.06-3.56) | 4.58 (4.26-4.90) | 3.58 (3.34-3.83) | 5.32 (4.99-5.64) | | 3.99 (3.72-4.25) | 6.34 (5.96-6.71) | |
| 90 | 6.02 (5.65-6.40) | 10.14 (9.55-10.73) | 6.07 (5.73-6.40) | 10.81 (10.26-10.36) | | 5.82 (5.50-6.14) | 11.15 (10.60-11.70) | |
| **Ischaemic stroke** |  |  |  |  | |  |  | |
| 40 | 0.01 (0-0.03) | 0.02 (0-0.03) | 0.02 (0-0.03) | 0.02 (0-0.03) | | 0.04 (0.02-0.07) | 0.06 (0.03-0.09) | |
| 50 | 0.06 (0.03-0.08) | 0.07 (0.04-0.09) | 0.07 (0.04-0.10) | 0.09 (0.05-0.12) | | 0.14 (0.10-0.19) | 0.19 (0.14-0.24) | |
| 60 | 0.16 (0.12-0.21) | 0.22 (0.16-0.27) | 0.21 (0.15-0.26) | 0.29 (0.22-0.35) | | 0.38 (0.30-0.46) | 0.53 (0.44-0.63) | |
| 70 | 0.49 (0.41-0.58) | 0.67 (0.57-0.78) | 0.60 (0.50-0.69) | 0.83 (0.71-0.94) | | 0.88 (0.76-1.01) | 1.40 (1.23-1.57) | |
| 80 | 1.41 (1.25-1.58) | 2.27 (2.04-2.50) | 1.47 (1.31-1.63) | 2.44 (2.21-2.66) | | 1.71 (1.53-1.88) | 3.29 (3.01-3.57) | |
| 90 | 2.92 (2.65-3.19) | 5.89 (5.42-6.37) | 2.87 (2.63-3.11) | 6.20 (5.76-6.64) | | 2.87 (2.64-3.09) | 6.93 (6.47-7.39) | |
| **Subarachnoid haemorrhage** |  |  |  |  | |  |  | |
| 40 | 0.03 (0.01-0.05) | 0.03 (0.01-0.05) | 0.04 (0.02-0.07) | 0.05 (0.02-0.07) | | 0.09 (0.05-0.12) | 0.09 (0.06-0.13) | |
| 50 | 0.13 (0.09-0.17) | 0.13 (0.09-0.17) | 0.13 (0.09-0.18) | 0.14 (0.10-0.19) | | 0.25 (0.19-0.31) | 0.27 (0.20-0.33) | |
| 60 | 0.27 (0.21-0.32) | 0.29 (0.23-0.35) | 0.28 (0.22-0.34) | 0.30 (0.24-0.37) | | 0.46 (0.37-0.54) | 0.51 (0.42-0.60) | |
| 70 | 0.36 (0.29-0.43) | 0.40 (0.33-0.48) | 0.48 (0.39-0.56) | 0.52 (0.43-0.61) | | 0.65 (0.55-0.75) | 0.74 (0.62-0.85) | |
| 80 | 0.47 (0.38-0.56) | 0.57 (0.47-0.67) | 0.60 (0.51-0.70) | 0.72 (0.61-0.84) | | 0.80 (0.68-0.91) | 1.02 (0.88-1.16) | |
| 90 | 0.54 (0.44-0.64) | 0.73 (0.59-0.86) | 0.68 (0.57-0.78) | 0.90 (0.76-1.05) | | 0.84 (0.72-0.96) | 1.17 (1.01-1.34) | |
| **Intracerebral haemorrhage** |  |  |  |  | |  |  | |
| 40 | 0.02 (0-0.04) | 0.02 (0-0.04) | 0 (0-0.01) | 0.01 (0-0.02) | | 0.04 (0.02-0.06) | 0.06 (0.03-0.09) | |
| 50 | 0.04 (0.02-0.07) | 0.04 (0.02-0.07) | 0.04 (0.01-0.06) | 0.05 (0.02-0.08) | | 0.08 (0.05-0.11) | 0.13 (0.08-0.17) | |
| 60 | 0.10 (0.07-0.14) | 0.12 (0.08-0.16) | 0.12 (0.08-0.16) | 0.16 (0.11-0.21) | | 0.20 (0.14-0.26) | 0.29 (0.22-0.36) | |
| 70 | 0.26 (0.20-0.33) | 0.34 (0.27-0.42) | 0.31 (0.24-0.38) | 0.41 (0.33-0.49) | | 0.43 (0.34-0.52) | 0.61 (0.50-0.72) | |
| 80 | 0.62 (0.51-0.73) | 0.92 (0.77-1.06) | 0.62 (0.52-0.73) | 1.03 (0.88-1.18) | | 0.78 (0.66-0.89) | 1.31 (1.14-1.49) | |
| 90 | 1.27 (1.09-1.45) | 2.25 (1.95-2.55) | 1.06 (0.92-1.21) | 2.16 (1.90-2.41) | | 1.12 (0.98-1.27) | 2.47 (2.19-2.75) | |
| **Peripheral arterial disease** |  |  |  |  | |  |  | |
| 40 | 0.05 (0.02-0.07) | 0.05 (0.02-0.08) | 0.07 (0.04-0.10) | 0.07 (0.04-0.10) | | 0.15 (0.11-0.20) | 0.16 (0.11-0.20) | |
| 50 | 0.13 (0.09-1.17) | 0.14 (0.10-0.18) | 0.22 (0.17-0.28) | 0.23 (0.18-0.29) | | 0.51 (0.42-0.59) | 0.55 (0.46-0.64) | |
| 60 | 0.38 (0.31-0.45) | 0.41 (0.34-0.48) | 0.59 (0.50-0.68) | 0.66 (0.56-0.76) | | 1.30 (1.16-1.45) | 1.58 (1.42-1.74) | |
| 70 | 1.12 (0.99-1.25) | 1.32 (1.17-1.47) | 1.54 (1.38-1.69) | 1.89 (1.72-2.07) | | 2.89 (2.66-3.11) | 3.77 (3.50-4.04) | |
| 80 | 2.71 (2.48-2.94) | 3.52 (3.245-3.79) | 3.35 (3.11-3.58) | 4.62 (4.32-4.93) | | 4.70 (4.41-4.98) | 6.96 (6.58-7.35) | |
| 90 | 4.27 (3.97-4.58) | 6.71 (6.24-7.19) | 4.82 (4.53-5.12) | 7.78 (7.33-8.24) | | 5.76 (5.44-6.08) | 9.85 (9.36-10.35) | |
| **Abdominal aortic aneurysm** |  |  |  |  | |  |  | |
| 40 | 0 (0-0.01) | 0 (0-0.01) | 0.01 (0-0.02) | 0.01 (0-0.02) | | 0 (0-0.01) | 0 (0-0.01) | |
| 50 | 0.01 (0-0.02) | 0.01 (0-0.02) | 0.02 (0-0.03) | 0.02 (0-0.03) | | 0.01 (0-0.03) | 0.02 (0-0.03) | |
| 60 | 0.03 (0.01-0.05) | 0.02 (0-0.04) | 0.02 (0-0.04) | 0.02 (0-0.04) | | 0.06 (0.03-0.09) | 0.05 (0.02-0.08) | |
| 70 | 0.09 (0.05 0.13) | 0.09 (0.05-0.14) | 0.11 (0.07-0.15) | 0.12 (0.08-0.17) | | 0.19 (0.13-0.25) | 0.26 (0.19-0.34) | |
| 80 | 0.47 (0.37-0.58) | 0.54 (0.42-0.66) | 0.48 (0.39-0.58) | 0.61 (0.49-0.72) | | 0.55 (0.45-0.65) | 0.80 (0.66-0.94) | |
| 90 | 0.99 (0.83-1.15) | 1.22 (1.00-1.44) | 0.94 (0.81-1.08) | 1.43 (1.22-1.64) | | 0.91 (0.78-1.04) | 1.59 (1.36-1.82) | |

Note: CA-SCD, atrial fibrillation, cardiac arrest and sudden cardiac death; CI, confidence interval; first event, first cardiovascular disease presentation of a specific type, regardless of prior occurrence of another type of cardiovascular disease; initial presentation, first presentation of cardiovascular disease of any type for a specific patient.
