# Supplementary material for: Socioeconomic Deprivation and the Incidence of 12 Cardiovascular Diseases in 1.9 Million Women and Men: Implications for Risk Prediction and Prevention
Source: PLoS One. 2014 Aug 21;9(8):e104671. doi: 10.1371/journal.pone.0104671 (PMC4140710; doi:10.1371/journal.pone.0104671)
Supplement: Table S2 — Lifetime risks of initial vs. first event presentation of twelve cardiovascular diseases by level of socioeconomic deprivation for consecutive attained ages in omen. Note: CA-SCD, atrial fibrillation, cardiac arrest and sudden cardiac death; CI, confidence interval; first event, first cardiovascular disease presentation of a specific type, regardless of prior occurrence of another type of cardiovascular disease; initial presentation, first presentation of cardiovascular disease of any type for a specific patient. (DOCX) [file pone.0104671.s017.docx]

**Table S2-1-2 Lifetime risk of initial vs. first event presentation of twelve cardiovascular diseases by level of socioeconomic deprivation for consecutive attained ages in men**

| **Age (years)** | **Least deprived (Q1)** | | **Q3** | | **Most deprived (Q5)** | |
| --- | --- | --- | --- | --- | --- | --- |
|  | **Percentage lifetime risk (95% CI)** | | **Percentage lifetime risk (95% CI)** | | **Percentage lifetime risk (95% CI)** | |
|  | **Initial presentation** | **First event** | **Initial presentation** | **First event** | **Initial presentation** | **First event** |
| **Stable angina** |  |  |  |  |  |  |
| 40 | 0.04 (0.02-0.07) | 0.12 (0.08-0.16) | 0.06 (0.03-0.09) | 0.17 (0.12-0.22) | 0.08 (0.05-0.11) | 0.29 (0.23-0.35) |
| 50 | 0.31 (0.25-0.37) | 0.97 (0.86-1.07) | 0.40 (0.33-0.47) | 1.34 (1.21-1.48) | 0.60 (0.5-0.69) | 2.13 (1.97-2.30) |
| 60 | 1.32 (1.19-1.44) | 3.99 (3.77-4.21) | 1.69 (1.54-1.84) | 5.13 (4.88-5.39) | 1.94 (1.77-2.11) | 6.76 (6.44-7.07) |
| 70 | 3.36 (3.14- 3.59) | 9.83 (9.45- 10.22) | 3.77 (3.54-4.01) | 11.12 (10.72- 11.52) | 4.03 (3.77-4.29) | 13.54 (13.06-14.02) |
| 80 | 5.48 (5.17-5.79) | 16.62 (16.05-17.18) | 5.97 (5.66-6.29) | 18.14 (17.57-18.70) | 5.59 (5.27-5.90) | 19.84 (19.22-20.46) |
| 90 | 6.55 (6.18-6.92) | 21.03 (20.27-21.79) | 6.96 (6.61-7.32) | 22.88 (22.15-23.61) | 6.10 (5.77-6.43) | 23.39 (22.65-24.12) |
| **Unstable angina** |  |  |  |  |  |  |
| 40 | 0.05 (0.03-0.08) | 0.08 (0.05-0.11) | 0.08 (0.04-0.11) | 0.10 (0.07-0.14) | 0.12 (0.09-0.16) | 0.20 (0.15-0.24) |
| 50 | 0.27 (0.21-0.32) | 0.45 (0.38-0.53) | 0.43 (0.35-0.50) | 0.67 (0.58-0.76) | 0.53 (0.45-0.61) | 1.12 (1.00-1.24) |
| 60 | 0.70 (0.60-0.79) | 1.39 (1.26-1.52) | 0.99 (0.88-1.11) | 1.99 (1.83-2.15) | 1.25 (1.11-1.38) | 3.00 (2.78-3.21) |
| 70 | 1.37 (1.23-1.51) | 3.25 (3.02-3.48) | 1.72 (1.56-1.87) | 3.89 (3.64-4.13) | 2.02 (1.84-2.19) | 5.30 (4.99-5.61) |
| 80 | 2.03 (1.85-2.22) | 5.67 (5.32-6.03) | 2.21 (2.02-2.39) | 6.27 (5.91-6.63) | 2.50 (2.30-2.71) | 7.78 (7.36-8.20) |
| 90 | 2.31 (2.09-2.52) | 7.80 (7.25-8.35) | 2.59 (2.37-2.80) | 8.80 (8.26-9.34) | 2.74 (2.52-2.95) | 9.71 (9.16-10.26) |
| **Myocardial infarction** |  |  |  |  |  |  |
| 40 | 0.10 (0.06-0.13) | 0.10 (0.07-0.14) | 0.16 (0.11-0.20) | 0.16 (0.12-0.21) | 0.26 (0.21-0.32) | 0.28 (0.22-0.33) |
| 50 | 0.71 (0.62-0.80) | 0.77 (0.67-0.86) | 0.90 (0.79-1.00) | 0.99 (0.87-1.10) | 1.47 (1.33-1.60) | 1.68 (1.53-1.82) |
| 60 | 2.35 (2.18-2.52) | 2.66 (2.48-2.84) | 2.81 (2.62-3.00) | 3.30 (3.09-3.51) | 3.92 (3.68-4.15) | 4.68 (4.41-4.94) |
| 70 | 4.76 (4.50-5.01) | 5.78 (5.48-6.08) | 5.33 (5.06-5.60) | 6.69 (6.37-7.01) | 6.56 (6.24-6.88) | 8.80 (8.40-9.19) |
| 80 | 7.12 (6.78-7.47) | 9.84 (9.39-10.29) | 8.17 (7.81-8.53) | 11.72 (11.24-12.20) | 8.97 (8.58-9.35) | 13.80 (13.5-14.34) |
| 90 | 9.10 (8.66-9.53) | 15.21 (14.44-15.98) | 10.10 (9.68-10.52) | 17.35 (16.62-18.09) | 10.29 (9.87-10.70) | 18.55 (17.80-19.30) |
| **Unheralded coronary death** |  |  |  |  |  |  |
| 40 | 0.02 (0-0.03) | 0.02 (0-0.04) | 0.02 (0-0.03) | 0.02 (0-0.03) | 0.04 (0.02-0.06) | 0.06 (0.03-0.08) |
| 50 | 0.10 (0.07-0.14) | 0.13 (0.09-0.17) | 0.14 (0.10-0.19) | 0.18 (0.13-0.23) | 0.28 (0.22-0.34) | 0.40 (0.33-0.47) |
| 60 | 0.33 (0.27-0.40) | 0.48 (0.40-0.56) | 0.45 (0.37-0.53) | 0.68 (0.58-0.78) | 0.85 (0.74-0.96) | 1.38 (1.24-1.53) |
| 70 | 0.85 (0.74-0.97) | 1.40 (1.25-1.56) | 0.97 (0.85-1.09) | 1.77 (1.60-1.94) | 1.79 (1.62-1.97) | 3.54 (3.27-3.81) |
| 80 | 1.78 (1.59-1.97) | 4.02 (3.68-4.35) | 1.88 (1.70-2.07) | 4.57 (4.23-4.92) | 2.82 (2.60-3.05) | 7.47 (7.02-7.93) |
| 90 | 2.97 (2.68-3.26) | 9.82 (9.04-10.60) | 2.93 (2.68-3.19) | 11.48 (10.71-12.25) | 3.80 (3.52-4.07) | 14.02 (13.22-14.82) |
| **CA-SCD** |  |  |  |  |  |  |
| 40 | 0.06 (0.02-0.09) | 0.05 (0.03-0.08) | 0.06 (0.03-0.09) | 0.05 (0.03-0.08) | 0.05 (0.03-0.08) | 0.04 (0.02-0.06) |
| 50 | 0.13 (0.09-0.18) | 0.14 (0.10-0.19) | 0.18 (0.13-0.23) | 0.19 (0.14-0.24) | 0.18 (0.13-0.23) | 0.17 (0.12-0.21) |
| 60 | 0.44 (0.36-0.51) | 0.51 (0.43-0.59) | 0.47 (0.39-0.55) | 0.59 (0.50-0.68) | 0.47 (0.38-0.55) | 0.65 (0.54-0.75) |
| 70 | 1.18 (1.04-1.32) | 1.63 (1.46-1.80) | 1.04 (0.92-1.17) | 1.53 (1.37-1.69) | 0.97 (0.84-1.09) | 1.53 (1.36-1.71) |
| 80 | 2.07 (1.87-2.27) | 3.44 (3.15-3.74) | 1.70 (1.53-1.87) | 3.00 (2.73-3.26) | 1.40 (1.24-1.56) | 2.54 (2.28-2.79) |
| 90 | 2.43 (2.20-2.66) | 4.42 (4.01-4.82) | 1.97 (1.78-2.16) | 4.11 (3.72-4.49) | 1.59 (1.42-1.77) | 3.35 (2.99-3.71) |
| **Heart failure** |  |  |  |  |  |  |
| 40 | 0.02 (0-0.03) | 0.01 (0-0.03) | 0.02 (0.01-0.04) | 0.03 (0.01-0.06) | 0.06 (0.03-0.08) | 0.07 (0.04-0.09) |
| 50 | 0.10 (0.07-0.13) | 0.11 (0.08-0.15) | 0.14 (0.10-0.19) | 0.19 (0.14-0.24) | 0.25 (0.19-0.31) | 0.32 (0.25-0.38) |
| 60 | 0.33 (0.26-0.39) | 0.44 (0.37-0.52) | 0.54 (0.46-0.63) | 0.84 (0.73-0.94) | 0.87 (0.76-0.99) | 1.27 (1.13-1.41) |
| 70 | 1.09 (0.96-1.22) | 1.69 (1.51-1.86) | 1.51 (1.36-1.66) | 2.46 (2.25-2.67) | 2.28 (2.08-2.48) | 3.82 (3.54-4.11) |
| 80 | 3.12 (2.86-3.37) | 5.77 (5.36-6.17) | 3.95 (3.68-4.22) | 7.49 (7.05-7.92) | 4.71 (4.42-5.01) | 9.83 (9.30-10.36) |
| 90 | 6.41 (5.97-6.85) | 14.43 (13.52-15.34) | 7.34 (6.93-7.75) | 17.94 (17.04-18.85) | 7.12 (6.74-7.50) | 18.72 (17.81-19.62) |
| **Transient ischaemic attack** |  |  |  |  |  |  |
| 40 | 0.03 (0.01-0.05) | 0.03 (0.01-0.06) | 0.02 (0.01-0.04) | 0.02 (0.01-0.04) | 0.05 (0.02-0.07) | 0.06 (0.04-0.09) |
| 50 | 0.15 (0.11-0.20) | 0.17 (0.13-0.22) | 0.15 (0.10-0.19) | 0.18 (0.14-0.23) | 0.26 (0.20-0.32) | 0.39 (0.32-0.47) |
| 60 | 0.60 (0.51-0.69) | 0.70 (0.61-0.80) | 0.69 (0.59-0.78) | 0.90 (0.79-1.01) | 0.87 (0.75-0.98) | 1.29 (1.15-1.43) |
| 70 | 1.74 (1.58-1.91) | 2.36 (2.16-2.57) | 1.95 (1.78-2.12) | 2.82 (2.59-3.04) | 2.10 (1.91-2.29) | 3.36 (3.10-3.63) |
| 80 | 3.62 (3.36-3.89) | 5.69 (5.30-6.07) | 3.90 (3.64-4.17) | 6.60 (6.21-7.01) | 3.74 (3.48-4.00) | 7.22 (6.78-7.67) |
| 90 | 5.75 (5.36-6.14) | 11.19 (10.44-11.95) | 5.59 (5.25-5.93) | 11.35 (10.68-12.01) | 4.89 (4.58-5.20) | 11.45 (10.76-12.15) |
| **Ischaemic stroke** |  |  |  |  |  |  |
| 40 | 0.02 (0-0.04) | 0.02 (0-0.4) | 0.02 (0-0.04) | 0.02 (0.01-0.04) | 0.05 (0.02-0.07) | 0.06 (0.03-0.08) |
| 50 | 0.10 (0.07-0.14) | 0.12 (0.08-0.16) | 0.09 (0.05-0.12) | 0.13 (0.09-0.17) | 0.20 (0.15-0.25) | 0.27 (0.21-0.32) |
| 60 | 0.32 (0.26-0.38) | 0.43 (0.35-0.50) | 0.35 (0.28-0.41) | 0.50 (0.41-0.58) | 0.58 (0.49-0.67) | 0.82 (0.71-0.94) |
| 70 | 0.79 (0.68-0.90) | 1.20 (1.05-1.35) | 0.91 (0.79-1.02) | 1.39 (1.23-1.55) | 1.32 (1.17-1.47) | 2.20 (1.99-2.41) |
| 80 | 1.67 (1.49-1.85) | 3.04 (2.76-3.33) | 1.91 (1.73-2.10) | 3.64 (3.34-3.95) | 2.12 (1.92-2.32) | 4.51 (4.15-4.86) |
| 90 | 2.95 (2.66-3.24) | 7.21 (6.54-7.87) | 2.99 (2.73-3.24) | 7.81 (7.18-8.43) | 2.93 (2.69-3.18) | 7.88 (7.28-7.48) |
| **Subarachnoid haemorrhage** |  |  |  |  |  |  |
| 40 | 0.2 (0-0.03) | 0.02 (0-0.04) | 0.02 (0-0.03) | 0.02 (0.01-0.04) | 0.04 (0.02-0.06) | 0.05 (0.03-0.08) |
| 50 | 0.10 (0.6-0.13) | 0.06 (0.03-0.09) | 0.11 (0.07-0.15) | 0.11 (0.07-0.14) | 0.20 (0.15-0.25) | 0.15 (0.11-0.19) |
| 60 | 0.35 (0.28-0.42) | 0.16 (0.11-0.20) | 0.50 (0.42-0.58) | 0.26 (0.20-0.32) | 0.78 (0.67-0.88) | 0.45 (0.36-0.53) |
| 70 | 0.94 (0.82-1.06) | 0.47 (0.38-0.56) | 1.32 (1.17-1.46) | 0.65 (0.55-0.76) | 1.69 (1.52-1.86) | 1.00 (0.86-1.14) |
| 80 | 2.12 (1.91-2.332) | 1.23 (1.04-1.42) | 2.88 (2.65-3.11) | 1.56 (1.36-1.76) | 3.05 (2.81-3.29) | 1.85 (1.62-2.08) |
| 90 | 3.71 (3.38-4.03) | 2.53 (2.12-2.93) | 4.50 (4.18-4.82) | 2.76 (2.40-3.11) | 4.24 (3.94-4.53) | 2.63 (2.31-2.96) |
| **Intracerebral haemorrhage** |  |  |  |  |  |  |
| 40 | 0.2 (0-0.4) | 0.02 (0-0.03) | 0.02 (0-0.03) | 0.03 (0.01-0.05) | 0.05 (0.02-0.07) | 0.02 (0.01-0.04) |
| 50 | 0.6 (0.3-0.9) | 0.07 (0.04-0.10) | 0.08 (0.04-0.11) | 0.11 (0.07-0.14) | 0.11 (0.08-0.15) | 0.14 (0.10-0.18) |
| 60 | 0.13 (0.9-0.17) | 0.15 (0.11-0.20) | 0.18 (0.13-0.23) | 0.20 (0.15-0.26) | 0.30 (0.23-0.36) | 0.31 (0.24-0.37) |
| 70 | 0.34 (0.27-0.41) | 0.31 (0.24-0.38) | 0.45 (0.36-0.53) | 0.29 (0.23-0.36) | 0.58 (0.48-0.68) | 0.48 (0.39-0.57) |
| 80 | 0.71 (0.59-0.83) | 0.44 (0.34-0.54) | 0.84 (0.72-0.96) | 0.42 (0.33-0.51) | 0.92 (0.79-1.05) | 0.60 (0.49-0.72) |
| 90 | 1.05 (0.88-1.22) | 0.58 (0.43-0.73) | 1.16 (1.01-1.32) | 0.54 (0.41-0.66) | 1.07 (0.93-1.21) | 0.67 (0.54-0.80) |
| **Peripheral arterial disease** |  |  |  |  |  |  |
| 40 | 0.04 (0.02-0.07) | 0.05 (0.03-0.08) | 0.05 (0.03-0.08) | 0.05 (0.02-0.07) | 0.11 (0.07-0.14) | 0.11 (0.08-0.15) |
| 50 | 0.20 (0.15-0.24) | 0.22 (0.17-0.27) | 0.24 (0.18-0.29) | 0.25 (0.20-0.31) | 0.57 (0.49-0.66) | 0.66 (0.57-0.75) |
| 60 | 0.72 (0.63-0.81) | 0.87 (0.76-0.97) | 0.96 (0.84-1.07) | 1.20 (1.07-1.33) | 2.23 (2.05-2.41) | 2.78 (2.57-2.99) |
| 70 | 1.81 (1.65-1.98) | 2.45 (2.24-2.65) | 2.53 (2.34-2.73) | 3.50 (3.26-3.75) | 4.75 (4.47-5.03) | 6.74 (6.37-7.10) |
| 80 | 3.31 (3.06-3.56) | 5.13 (4.78-5.49) | 4.49 (4.21-4.77) | 7.30 (6.89-7.71) | 6.89 (6.54-7.23) | 11.30 (10.78-11.82) |
| 90 | 4.56 (4.23-4.90) | 8.60 (7.97-9.22) | 5.71 (5.38-6.04) | 10.91 (10.29-11.53) | 7.80 (7.43-8.18) | 14.70 (14.01-15.39) |
| **Abdominal aortic aneurysm** |  |  |  |  |  |  |
| 40 | 0.01 (0-0.02) | 0 (0-0.01) | 0.01 (0-0.02) | 0.01 (0-0.02) | 0 (0-0.01) | 0.01 (0-0.01) |
| 50 | 0.02 (0.01-0.04) | 0.01 (0-0.02) | 0.03 (0.01-0.05) | 0.03 (0.01-0.05) | 0.01 (0-0.03) | 0.02 (0-0.03) |
| 60 | 0.08 (0.05-0.11) | 0.08 (0.05-0.11) | 0.11 (0.07-0.15) | 0.11 (0.07-0.15) | 0.12 (0.08-0.16) | 0.17 (0.12-0.22) |
| 70 | 0.59 (0.49-0.69) | 0.75 (0.63-0.87) | 0.58 (0.48-0.68) | 0.85 (0.72-0.98) | 0.57 (0.47-0.68) | 0.95 (0.80-1.11) |
| 80 | 1.65 (1.46-1.84) | 2.60 (2.32-2.88) | 1.64 (1.47-1.82) | 2.93 (2.64-3.22) | 1.53 (1.35-1.70) | 3.01 (2.70-3.32) |
| 90 | 2.82 (2.53-3.11) | 5.73 (5.14-6.32) | 2.50 (2.26-2.74) | 5.36 (4.87-5.85) | 2.07 (1.86-2.28) | 4.89 (4.41-5.37) |

Note: CA-SCD, atrial fibrillation, cardiac arrest and sudden cardiac death; CI, confidence interval; first event, first cardiovascular disease presentation of a specific type, regardless of prior occurrence of another type of cardiovascular disease; initial presentation, first presentation of cardiovascular disease of any type for a specific patient.
