# Supplementary material for: Socioeconomic Deprivation and the Incidence of 12 Cardiovascular Diseases in 1.9 Million Women and Men: Implications for Risk Prediction and Prevention
Source: PLoS One. 2014 Aug 21;9(8):e104671. doi: 10.1371/journal.pone.0104671 (PMC4140710; doi:10.1371/journal.pone.0104671)
Supplement: Text S1 — CALIBER program: study data sources. (DOCX) [file pone.0104671.s018.docx]

**Text 1.1 CALIBER program: study data sources**

The CALIBER (Cardiovascular disease research using Linked Bespoke studies and Electronic health Records) program was conceived to gain understanding of the aetiology and prognosis of specific coronary phenotypes[^1^](#_ENREF_10). Electronic medical records from patients registered in 225 general practices were linked across four data sources: the Clinical Practice Research Datalink (CPRD)^2^; the Myocardial Ischaemia National Audit Project registry (MINAP)^3^; Hospital Episodes Statistics (HES); and the Office of National Statistics (ONS). CPRD provides primary care data on health behaviours, diagnoses, investigations, procedures and prescriptions; and its accuracy and completeness are regularly audited. MINAP is a national registry of patients hospitalised with acute coronary syndromes in England and Wales. HES provides information on all hospital admissions and ONS cause-specific mortality records for all deaths in England and Wales. Information is coded using the hierarchical clinical coding schemes (Read[^4^](#_ENREF_11), the International Statistical Classification of Diseases and Health Related Problems, 10th revision[^5^](#_ENREF_12), and Office of the Population Censuses and Surveys Classification of Interventions and Procedures[^6^](#_ENREF_13), 4^th^ revision).

**Reference List**

1. Denaxas SC, George J, Herrett E, Shah AD, Kalra D, Hingorani AD, et al. Data resource profile: cardiovascular disease research using linked bespoke studies and electronic health records (CALIBER). Int J Epidemiol. 2012; **41**: 1625-38.
2. Walley T, Mantgani A. The UK General Practice Research Database. Lancet. 1997; **350**: 1097-1099.
3. Herrett E, Smeeth L, Walker L, Weston C. The Myocardial Ischaemia National Audit Project (MINAP). Heart. 2010; **96**(16):1264-7.
4. Chisholm J. The Read clinical classification. BMJ. 1990; **300**: 1092.
5. International Statistical Classification of Diseases and Related Health Problems, Tenth Revision (ICD-10). Vols 1-3. Geneva, World Health Organization, 1992-2000. Fourth Edition.
6. OPCS-4 Classification - NHS Connecting for Health; 2013.
