# Supplementary material for: Socioeconomic Deprivation and the Incidence of 12 Cardiovascular Diseases in 1.9 Million Women and Men: Implications for Risk Prediction and Prevention
Source: PLoS One. 2014 Aug 21;9(8):e104671. doi: 10.1371/journal.pone.0104671 (PMC4140710; doi:10.1371/journal.pone.0104671)
Supplement: Text S2 — Multiple imputation. (DOCX) [file pone.0104671.s019.docx]

**Text 1-3 Multiple imputation**

Baseline smoking status and risk factor data appeared to be missing at random after adjusting for major confounders (e.g. age, sex, diabetes, BMI and blood pressure). Hence, multiple imputation was implemented using the *mice*^1^ algorithm in the statistical package R, to replace missing values in exposure and risk factor variables. Imputation models were estimated separately for men and women and included:

1. all the baseline covariates used in the main analysis (age, quadratic age, diabetes, smoking, systolic blood pressure, total cholesterol, HDL cholesterol, index of multiple deprivation);
2. prior (between 1 and 4 years before study entry) and post (between 0 and 1 year after study entry) averages of continuous covariates in the main analysis;
3. baseline measurements of covariates not considered in the main analysis (diastolic blood pressure, alcohol intake, white cell count, haemoglobin, creatinine, alanine transferase);
4. baseline medications (statins, blood pressure lowering medication, low-dose aspirin, loop diuretics, oral contraceptives and hormone replacement therapy);
5. coexisting medical conditions (history of depression, cancer, renal disease, liver disease and chronic obstructive pulmonary disease);
6. the Nelson-Aalen hazard and the event status for each of the 12 endpoints analysed^2^.

Non-normally distributed variables were log-transformed for imputation and exponentiated back to their original scale for analysis. Five multiply imputed datasets were generated, and Cox models were fitted to each dataset. Coefficients were combined using Rubin’s rules. The Kolmogorov-Smirnov test was used to compare the distribution of observed versus imputed log-transformed covariates.

**Reference List**

1. van Buuren S. Multiple imputation of discrete and continuous data by fully conditional specification. Stat Methods Med Res. 2007; **16**: 219-242.
2. White IR, Royston P. Imputing missing covariate values for the Cox model. Stat Med. 2009; **28**: 1982-1998.
